# Reduced Health-Related Quality of Life in Body Constitutions of Yin-Xu, and Yang-Xu, Stasis in Patients with Type 2 Diabetes: Taichung Diabetic Body Constitution Study

**DOI:** 10.1155/2014/309403

**Published:** 2014-06-29

**Authors:** Chia-I Tsai, Yi-Chang Su, Shih-Yi Lin, I-Te Lee, Cheng-Hung Lee, Tsai-Chung Li

**Affiliations:** ^1^Graduate Institute of Chinese Medicine, College of Chinese Medicine, China Medical University, Taichung 40402, Taiwan; ^2^Department of Traditional Chinese Medicine, Taichung Veterans General Hospital, Taichung 40402, Taiwan; ^3^School of Chinese Medicine, College of Chinese Medicine, China Medical University, 91 Hsueh-Shih Road, Taichung 40402, Taiwan; ^4^Division of Endocrinology and Metabolism, Department of Internal Medicine, Taichung Veterans General Hospital, Taichung 40705, Taiwan; ^5^Institute of Medicine, Chung Shan Medical University, Taichung 40201, Taiwan; ^6^School of Medicine, National Yang-Ming University, Taipei 11221, Taiwan; ^7^Graduate Institute of Biostatistics, College of Management, China Medical University, 91 Hsueh-Shih Road, Taichung 40402, Taiwan; ^8^Research Center for Chinese Medicine & Acupuncture, China Medical University, 91 Hsueh-Shih Road, Taichung 40402, Taiwan; ^9^Department of Healthcare Administration, College of Health Science, Asia University, Taichung 41354, Taiwan

## Abstract

*Aim*. To evaluate how health-related quality of life (HRQOL) and traditional Chinese medicine (TCM) constitutions of Yin-Xu, Yang-Xu, and Stasis are related in type 2 diabetes patients. *Method*. Seven hundred and five subjects were recruited in 2010 for this study from a Diabetes Shared Care Network in Taiwan. Generic and disease-specific HRQOL were assessed by the short form 36 (SF-36) and the diabetes impact measurement scale (DIMS). Constitutions of Yin-Xu, Yang-Xu, and Stasis were then assessed by the body constitution questionnaire (BCQ), a questionnaire consisting of 44 items that evaluate the physiological state based on subjective symptoms and signs. *Results*. Estimated effects of the Ying-Xu and Stasis on all scales of the SF-36 were significantly negative, while estimated effects of the Yang-Xu on all scales (except for SF, RE, MH, and MCS) were significantly negative. For DIMS, the estimated effects of the Ying-Xu and Stasis on all scales were significantly negative except for Stasis on well-being, while Yang-Xu has a significantly negative effect only on symptoms. *Conclusions*. This study demonstrates that TCM constitutions of Yin-Xu, Yang-Xu, and Stasis are closely related to a reduction in HRQOL. These findings support the need for further research into the impact of intervention for TCM constitutions on HRQOL in patients with type 2 diabetes.

## 1. Introduction

Diabetes in all age groups worldwide has significantly increased in recent decades [1–4]. The rapidly growing incidence and prevalence of diabetes severely strains health care systems in terms of managing the disease and its related complications [5, 6]. Diabetics encounter many challenges when attempting to control this disease, adversely impacting quality of life [7]. As advances in medical care have prolonged life, elevating the quality of life is essential for medical treatment [8]. Health care for such patients focuses mainly on maximizing daily life functions and achieving the highest level of well-being may reduce health care costs [9]. Health-related quality of life (HRQOL) is an essential outcome of medical care, in addition to disease-specific measures [10]. The short form 36 (SF-36) and diabetes impact measurement scale (DIMS) evaluate health concepts that represent basic human values relevant to the functional status and well-being of diabetics [11].

The constitution theory not only serves as an important foundation of clinical TCM practice but also has been applied for more than 2,000 years to evaluate patients in states of subhealth, subdisease, or predisease [12]. However, exactly how HRQOL and TCM body constitutions are related has seldom been studied. Therefore, this study adopts the body constitution questionnaire (BCQ), a well-established instrument.

Yin-Xu (Yin deficiency) constitution implies that individuals' materials to perform or maintain body functions have diminished, and they may experience some subjective symptoms (e.g., thirst, hot flush, hard stool, and small volume of urine) [13, 14]. Yang-Xu (Yang deficiency) constitution implies a diminishing energy level in the physiological functioning of the body, and individuals may experience symptoms (e.g., fatigue, shortness of breath, chills, loose stool, and a large volume of urine) [15, 16]. Stasis constitution implies that individuals' dynamic interaction between Yin and Yang is decelerated and less efficient. These individuals may express some physical symptoms (e.g., dizziness, chest tightness, and numbness in the limbs) [17]. These are the three major TCM body constitutions applied in the TCM treatment.

Several studies have demonstrated that TCM treatment is promising for treating diabetes [18–21]. This treatment is a viable alternative for diabetics attempt to improve their quality of life. Additionally, some studies of the National Health Insurance Research database have revealed that the use of TCM for treating diabetes is popular in Taiwan [22–25], but no study of the related diagnostic basis for its use has been examined. Although previous research has examined how these TCM body constitutions affect pregnant women by using the instrument of BCQ, to our knowledge, no study has been performed on type 2 diabetics [26]. No investigation of the relationship between HRQOL and TCM body constitutions has been investigated. Therefore, this cross-sectional study is the first to examine the domains and degrees of functioning and well-being that are affected by the TCM body constitutions of Yin-Xu, Yang-Xu, and Stasis of type 2 diabetics cared for in the Diabetes Shared Care Network (DSCN) in Taiwan.

## 2. Materials and Methods

### 2.1. Participants

The study participants were men and women over 18 years old and were recruited between February 2010 and February 2011 from the Diabetes Shared Care Network of the Taichung Veterans General Hospital (Taichung, Taiwan). All participants had a diagnosis of type 2 DM, based on the criteria of the American Diabetes Association, and had fasting plasma >126 mg/dL, or two-hour plasma glucose >200 mg/dL during oral glucose tolerance test (OGTT) on two separate occasions, or random plasma glucose >200 mg/dL with symptoms (polyuria, polydipsia, and unexplained weight loss). Patients who had taken Chinese medicine within the past month were excluded from the study. This study complied with the Declaration of Helsinki. The relevant institutional ethics review boards approved the protocol, and all participants provided informed consent.

### 2.2. Protocol

DSCN in Taiwan is a team management program for diabetics that is supported by the Taiwan National Health Insurance (NHI). Patients are invited to participate after an endocrinologist diagnoses them with DM; they receive regular relevant medical and examinations quarterly [27]. The databank maintained information on changes in diabetes-related information concerning the patients, including their gender, age, duration of illness, HbA1c, blood pressure (BP), proteinuria, renal function, blood lipid profiles, and the results of ocular and foot examinations. Physical and biochemical examinations were performed within one month of the self-administration of SF-36, DIMS, and BCQ. Physical and biochemical markers were measured and their correlations with HRQOL and TCM constitutions in type 2 diabetes patients were analyzed, using the results of a Taichung diabetic body constitution study (TDBS) of type 2 diabetics who were cared for in the DSCN in Taichung, Taiwan.

### 2.3. Measurements

#### 2.3.1. Body Constitution Questionnaire (BCQ)

The physiological state of constitution was measured by BCQ, which was developed by Su. The questionnaire consists of 44 items of a 5-point Likert-type response scale (from 1 (never happened) to 5 (always happens)) with a total score ranging from 44 to 220. All items were organized into three independent constitution scales, including 19 items in Yang deficiency with the scores ranging from 19 to 95; 19 items in Yin deficiency with scores ranging from 19 to 95; and 16 items in Stasis with scores ranging from 16 to 80. Some items belonging to these three scales overlapped each other. A higher score implies a greater deviation of the constitution. According to a previous study BCQ has a good factorial validity [28]. In previous studies, Cronbach's *α* of each constitution subscale ranged from 0.85 to 0.88 [14, 16, 17]. The overall Cronbach's alpha of BCQ was 0.90 [29]. In this study, Cronbach's *α* ranged from 0.67 to 0.70. The test-retest reliability of BCQ was measured by Spearman correlation coefficient (*r*). All *r* values of subscales of Yang-Xu body constitution questionnaire (BCQ+), Yin-Xu body constitution questionnaire (BCQ−), and Stasis body constitution questionnaire (BCQs) were 0.84 [14, 16, 17]. The content validity of the items of BCQ was evaluated through a Delphi process with a CVI value of ≥0.7 [13, 15]. Construct validity was assessed by exploratory factor analyses (EFA). The factors were extracted by principal component analysis (PCA) with Promax oblique rotation and determined by the criteria of the eigenvalue >1. Student's *t*-test was used to compare consistency between the BCQ score and the diagnosis by the TCM physician [16] for construct validity. The evaluation of convergent validity had been reported by Wang et al. [30]. The correlation coefficients between the 5 factors and the total scale were 0.77, 0.82, 0.73, 0.69, and 0.54, respectively, representing significant correlations (*P* < 0.0001) that indicate BCQ+ has good convergent validity.

#### 2.3.2. Short Form 36 (SF-36)

The SF-36 contains 36 items which measure eight multi-item variables: physical functioning (PF, 10 items), social functioning (SF, 2 items), role limitations due to physical problems (RP, 4 items), role limitations due to emotional problems (RE, 3 items), mental health (MH, 5 items), vitality (VT, 4 items), pain (BP, 2 items), and general perception of health (GH, 5 items). For each variable item, scores are coded, summed, and transformed to a scale from 0 (worst possible health state measured by the questionnaire) to 100 (best possible health state). Additionally, the SF-36 Physical Component Summary (PCS) and the Mental Component Summary (MCS) scales are derived following the standard SF-36 scoring algorithms [31]. For the SF-36, a high score implies a better state of health [32].

#### 2.3.3. Diabetes Impact Measurement Scale (DIMS)

DIMS consisted of 44 items measuring four domains: symptoms (17 items), well-being (11 items), diabetes-related morale (patient attitude towards managing the disease, 11 items), and social role fulfillment (five items). DIMS required approximately 15–20 minutes to complete. All items were scored on a scale of zero-four in relation to their frequency of occurrence during the past month (0 = all of the time and 4 = none of the time). Items were scored based on the patient's response, with high values representing less severe or less frequent symptoms, greater morale, greater social role fulfillment, and greater well-being. An overall score was obtained by summing up the responses across items in the same scale. During the pretest period, 20 diabetic patients were retested with the same questionnaire 1-2 weeks after its mailing to all patients. The Pearson's correlation coefficients ranged from moderate to high for test-retest coefficients: symptoms (0.55), morale (0.78), social role fulfillment (0.76), well-being (0.79), and total score (0.92) among the nine patients who responded that they discern no change in the overall quality of life during the one-week period immediately before administering the second round of the questionnaire [33].

### 2.4. Statistical Analysis

Data were analyzed when appropriate by using simple descriptive analyses, such as mean, standard deviation, proportion, Chi-square test, and *t*-test. Individuals with and without Yin-Xu, Yang-Xu, and Stasis were compared in terms of global group differences in SF-36 scales by using a two-sample *t*-test.

This study also examined the relative burden of Yin-Xu, Yang-Xu, and Stasis on the scales by comparing the *t*-values of two-sample *t*-tests across eight scales. This assessment method was based on statistical efficiency [34, 35]. A scale is more efficient than another one if it yields a higher ratio of systematic variation than that of random variation. When the sample size is maintained constant within comparisons of eight scales, the relative precision of these scales can be detected by comparing the magnitudes of the *F* statistic (ratio of systematic variance relative to error variance) [36]. For a two-sample *t*-test, the square of *t*-value is equivalent to the *F* statistic.

By using the multiple linear regression model, the independent effects of Yin-Xu, Yang-Xu, and Stasis on physical functioning and well-being were evaluated by controlling the other independent variables. Regression models estimated the extent to which Yin-Xu, Yang-Xu, and Stasis affect HRQOL (SF-36 and DIMS) by comparing patients with Yin-Xu, Yang-Xu, or Stasis to diabetics screened as not having these constitutions.

## 3. Results

The study group was composed of 705 type 2 diabetics cared for in the DSCN in Taichung City, with an average age of 63.90 years (standard deviation, SD = 13.35 years). More than half of the study group was male (*n* = 405, 57.5%). The duration of diabetes and BMI were 9.03 years (SD = 8.12 years) and 25.46 kg/m^2^ (SD = 4.01 kg/m^2^). Of the 705 participants, 196 (27.80%), 91 (12.91%), and 91 (12.91%) were categorized as Yin-Xu, Yang-Xu, and Stasis, respectively.  Table 1 compares the demographic factors, chronic disease/conditions, and biomarkers between patients with and without Yin-Xu, Yang-Xu, and Stasis. Patients with Yin-Xu were older and with a lower level of eGFR than the others, and patients with Stasis were with higher mean values of BMI, waist, HbA1c, and LDL cholesterol than the others.

Table 2 lists the means and standard deviations of scores for SF-36 and DIMS scales, according to constitutions of Yin-Xu, Yang-Xu, and Stasis. Patients with Yin-Xu, Yang-Xu, and Stasis generally reported a significantly worse health condition than those without counterpart constitutions on all scales of SF-36 and DIMS. The significantly negative differences of SF-36 between patients with and without Yin-Xu ranged from 2.62 points for MCS to 21.96 points for RP, and differences of DIMS ranged from 1.57 points for the well-being scale to 6.16 points for the symptom scale. The significantly negative differences of SF-36 between patients with and without Yang-Xu ranged from 3.52 points for MCS to 27.12 points for RP, and differences of DIMS ranged from 1.24 points for the well-being scale to 6.44 points for the symptom scale. The significantly negative differences of SF-36 between patients with and without Stasis ranged from 4.97 points for MCS to 33.83 points for RP, and differences of DIMS ranged from 2.33 points for the well-being scale to 6.35 points for the symptom scale.

The scale with the largest value of the *F* statistic indicated this scale had the best ability to discriminate between patients with type 2 diabetes in the “yes” and “no” response categories of body constitution among these scales. For SF-36, the *F* statistics associated with the differences between the adjusted mean scores of diabetic patients with and without the Yin-Xu constitution were highest for the GH scale (*F* statistic = 66.16) and lowest for the MCS scale (*F* statistic = 14.59). Similarly, the *F* statistic for the Yang-Xu constitution was highest for the VT scale (*F* statistic = 58.98) and lowest for the MCS scale (*F* statistic = 15.05). The *F* statistic for the Stasis constitution was highest for the VT scale (*F* statistic = 99.60) and lowest for the RE scale (*F* statistic = 22.94). These results reveal that the GH scale is better able than any other SF-36 scale to distinguish diabetic patients with the Yin-Xu constitution from those without it. The scale with the best ability to distinguish Yang-Yu from Stasis constitutions was the VT scale. For DIMS, the *F* statistics of the differences between the adjusted mean scores of diabetic patients with and without the Yin-Xu constitution were highest for the symptom scale (*F* statistic = 226.80) and lowest for the social role fulfillment scale (*F* statistic = 22.18). Similarly, the *F* statistics for the Yang-Xu constitution were highest for the symptom scale (*F* statistic = 122.99) and lowest for the well-being scale (*F* statistic = 9.49). The *F* statistic for Stasis constitution was highest for the symptom scale (*F* statistic = 117.94) and lowest for the well-being scale (*F* statistic = 33.87). These results reveal that the symptom scale was superior to the other DIMS scales in its ability to distinguish between diabetic patients with Yin-Xu, Yang-Yu, or Stasis constitutions and those without the corresponding constitution.

Multiple regression analysis was performed to estimate simultaneously the effects of Yin-Xu, Yang-Xu, and Stasis on the eight scales and two summary scales of SF-36 using multivariate adjustment (Table 3). In general, the estimated effects of the Yin-Xu and Stasis on all scales of the SF-36 were significantly negative. Moreover, the estimated effects of the Yang-Xu on all scales (except for SF, RE, MH, and MCS) were significantly negative. The magnitude of the effects was generally the largest for Stasis and next for Yin-Xu. According to the standardized regression coefficients of Yin-Xu, Yang-Xu, and Stasis, Yin-Xu most significantly impacted BP, GH, RE, and PCS, which were scales of the primary physical component. Yang-Xu most significantly impacted the PF scale. Stasis most significantly impacted RP, VT, SF, MH, and MCS, which were scales of the primary mental component. Percentages of the variations of these 10 scales explained by these factors ranged from 16% to 41%, with the lowest percentage for RE and the highest percentage for PF.

Multiple regression analysis was performed to estimate simultaneously the effects of Yin-Xu, Yang-Xu, and Stasis on the four scales of DIMS using multivariate adjustment (Table 4). In general, the estimated effects of the Yin-Xu on all scales of DIMS were significantly negative and the estimated effects of the Stasis on all scales (except for well-being) were significantly negative. The only significantly negative scale of Yang-Xu was symptoms. The magnitude of the effects was generally the largest for Stasis and next for Yin-Xu. According to the standardized regression coefficients of Yin-Xu, Yang-Xu, and Stasis, Yin-Xu most significantly impacted symptoms and related morale. Yang-Xu most significantly impacted symptoms. Stasis most significantly impacted symptoms and related morale.

## 4. Discussion

Ayurvedic medicine is similar in certain respects to TCM. Both systems are basically aimed at improving health. In TCM, the Yin and Yang theory plays a prominent role, while Ayurveda emphasizes the supremacy of the three doshas (tridoshas) system [37]. In TCM, a possible suitable term to describe Pitta is fire. Kapha in Ayurveda has the characteristics of phlegm in TCM. The description for Vata in Ayurveda is similar to wind in TCM. These three items are explained as stages of transformation (following food transformation and transportation). Health is achieved through a balance of Yang and Yin, while Yang-Xu (Yang deficiency) implies a diminishing energy level in the physiological functioning of the body. Ying-Xu (Ying deficiency) implies a diminishing material level in the physiological functioning of the body. Stasis constitution implies that a person's dynamic interaction between Yin and Yang is slowed down and less efficient. It can be influenced by phlegm (or Kapha) [38]. From the viewpoint of anthroposophic medicine, an individual is influenced by different forces whose “higher ranking members” intervene in a regulatory manner in somatic processes [39]. In the context of anthroposophical medicine or homeopathy, constitution is central to diagnostic and treatment decisions [40]. In the theory of anthroposophic medicine, humans have four constitutions: physical body, etheric body, astral body, and body of the ego. Four constitutions and the spirit orientation comprise the elements of the human body (physical body and etheric body), heart (astral body), and spirit (body of the ego and spirit orientation). Good health can only be achieved by attaining a balance between body, heart, and soul. Some researchers believe that the etheric body and astral body can be viewed as Yin and Yang in TCM [39]. In traditional Chinese medicine (TCM), diabetes closely resembles Xiao-ke disease, which is characterized by excess eating, drinking, and urination. Its pathogenesis is characterized by deficiency of yin which is combined with heat. According to our study's findings, the proportions of patients with constitutions of Yin-Xu, Yang-Xu, Stasis were 27.80%, 12.91%, and 12.91%. These findings are consistent with the concepts of diabetes in TCM.

This descriptive and correlational study has evaluated how constitution impacts the functioning and well-being in type 2 diabetic patients. Patients with Yin-Xu, Yang-Xu, or Stasis constitutions report more significantly compromised HRQOL than those patients without these constitutions in the same population. This finding suggests that constitution significantly impacts not only the scales of the primary physical component but also the scales of the primary mental component. Most of the effects are statistically and clinically significant if one assumes that differences of three to five points are considered clinically meaningful [41]. According to our results, patients with Yin-Xu, Yang-Xu, or Stasis constitutions negatively affect the eight scales of SF-36 and the four scales of DIMS. Of these three constitutions, Stasis constitution generally exerts the greatest effect.

Our results further indicate that the reduction in HRQOL associated with Yin-Xu, Yang-Xu, or Stasis constitutions is higher in magnitude than that reported for chronic physical illnesses such as lower back pain, arthritis, diabetes [10], and frailty [42], thus implying the severe impact of these constitutions. For instance, the negative effect of Yang-Xu constitution on physical functioning in this study is −8.38 that is markedly worse than the impact of diabetes (−6.3) [10].

Despite its contributions, this study has several limitations. First, the cross-sectional design of this study does not allow for any prospective conclusion on the relationship of Yin-Xu, Yang-Xu, or Stasis constitutions with HRQOL. Second, the sample in this study is selected from a diabetic population of a medical center in central Taiwan. Our results may thus be inapplicable to those patients in other clinical settings. Last, due to the limitations of this study design, it was not possible to compare the results with those of a healthy control group. In future investigations we intend to use a study design that includes a healthy control group. Although we did not have a healthy control for comparison, the purpose of the study was to determine how the constitutions of type 2 diabetics were influenced by type 2 DM and this purpose can be answered without having a healthy control.

Despite the above limitations, this study examines for the first time how Yin-Xu, Yang-Xu, or Stasis constitutions impact the function and well-being in type 2 diabetics. In addition to measuring the functional status, well-being, and overall health, which are of primary concern to patients, SF-36 and DIMS also provide standards for HRQOL. This study also illustrates the profiles of HRQOL for patients with Yin-Xu, Yang-Xu, or Stasis constitutions, which are in contrast to those without. Elucidating the association between constitutions and HRQOL provides further insight into the differences between generic and disease-specific health measures scale scores and the clinical measures of symptoms and signs that are familiar to clinicians.

Results of this study demonstrate that the differences in HRQOL between patients with and without constitutions are substantial, and constitutions might account for the difference. We recommend that future studies explore the longitudinal relationship between constitutions. Moreover, longitudinal studies should be conducted to further elucidate the causal relationship between constitutions and HRQOL. BCQ is a promising alternative for guiding clinicians in using the proper indicators, combining them systematically, and generating conclusions regarding diabetes care management.

## 5. Conclusions

This study demonstrates the feasibility of linking the TCM body constitutions to a reduction in HRQOL, as measured by SF-36 and DIMS. Future studies are needed to assess if interventions improve TCM body constitutions of Yin-Xu, Yang-Xu, and Stasis and lead to improvement in HRQOL.

## Figures and Tables

**Table 1:** Comparisons of basic characteristics between constitutions of Yin-Xu, Yang-Xu, and Stasis.

Variable	Total (*n* = 705)	Yin-Xu (*n* = 705)			Yang-Xu (*n* = 705)			Stasis (*n* = 705)		
		No (*n* = 509)	Yes (*n* = 196)	*P* value	No (*n* = 614)	Yes (*n* = 91)	*P* value	No (*n* = 614)	Yes (*n* = 91)	*P* value
Gender										
Male, *n* (%)	405 (57.5%)	304 (43.1%)	101 (14.3%)	0.05	369 (52.3%)	36 (5.11%)	0.00∗∗	364 (51.6%)	41 (5.82%)	0.01∗
Age (yr)	63.90 ± 13.35	63.24 ± 13.24	65.61 ± 13.53	0.04∗	63.97 ± 13.21	63.45 ± 14.36	0.72	63.79 ± 13.29	64.69 ± 13.81	0.58
BMI (kg/m^2^)	25.46 ± 4.01	25.46 ± 4.15	25.46 ± 3.63	0.97	25.43 ± 3.99	25.67 ± 4.13	0.59	25.32 ± 3.98	26.45 ± 4.05	0.01∗
Duration of diabetes (years)	9.03 ± 8.12	8.74 ± 7.69	9.78 ± 9.14	0.15	8.86 ± 7.81	10.16 ± 9.95	0.21	9.05 ± 8.04	8.87 ± 8.72	0.78
Waist (cm)	89.10 ± 10.65	89.33 ± 10.88	88.52 ± 10.02	0.40	89.17 ± 10.48	88.63 ± 11.76	0.66	88.67 ± 10.44	92.07 ± 11.56	0.01∗
Smoking				0.87			0.88			0.13
No	691 (98.01%)	499 (70.78%)	192 (27.23%)		602 (85.39%)	89 (12.62%)		605 (85.82%)	86 (12.20%)	
Sometimes	9 (1.28%)	6 (0.85%)	3 (0.43%)		8 (1.13%)	1 (0.14%)		7 (0.99%)	2 (0.28%)	
Usually	5 (0.71%)	4 (0.57%)	1 (0.14%)		4 (0.57%)	1 (0.14%)		3 (0.43%)	2 (0.28%)	
Physical activity				0.09			0.02∗			0.004∗∗
No	149 (21.19%)	98 (13.94%)	51 (7.25%)		123 (17.50%)	26 (3.70%)		118 (16.79%)	31 (4.41%)	
Sometimes	197 (28.02%)	138 (19.63%)	59 (8.39%)		164 (23.33%)	33 (4.69%)		170 (24.18%)	27 (3.84%)	
Usually	357 (50.79%)	271 (38.55%)	86 (12.23%)		325 (46.23%)	32 (4.55%)		325 (46.23%)	32 (4.55%)	
GFR (mL/min)	67.37 ± 22.11	68.73 ± 21.44	63.91 ± 23.43	0.03∗	67.62 ± 21.12	65.75 ± 27.85	0.38	67.52 ± 21.38	66.35 ± 26.78	0.82
Microalbumin (mg/dL)	21.96 ± 69.64	19.42 ± 59.95	28.40 ± 89.47	0.19	20.86 ± 67.13	29.84 ± 87.44	0.41	20.81 ± 66.62	30.05 ± 87.98	0.34
Fasting glucose (mg/dL)	142.98 ± 48.20	142.34 ± 44.75	144.62 ± 56.17	0.51	141.82 ± 45.28	150.73 ± 64.15	0.18	142.14 ± 47.84	148.73 ± 50.54	0.15
HbA1c (%)	7.65 ± 1.58	7.59 ± 1.52	7.80 ± 1.71	0.11	7.61 ± 1.56	7.92 ± 1.70	0.08	7.58 ± 1.56	8.10 ± 1.66	0.004∗∗
Triglycerides (mg/dL)	151.99 ± 170.80	154.82 ± 195.41	144.80 ± 79.36	0.36	151.28 ± 177.51	156.68 ± 117.94	0.73	155.89 ± 177.56	152.66 ± 115.79	0.99
HDL-C (mg/dL)	52.02 ± 14.10	52.31 ± 14.44	51.26 ± 13.20	0.61	52.14 ± 14.24	51.18 ± 13.18	0.53	52.13 ± 14.16	51.20 ± 13.71	0.85
LDL-C (mg/dL)	106.26 ± 31.33	105.39 ± 30.23	108.52 ± 34.04	0.39	105.90 ± 31.43	108.76 ± 30.75	0.31	105.26 ± 30.73	113.58 ± 34.79	0.04∗
SBP (mmHg)	131.35 ± 14.67	131.38 ± 14.56	131.30 ± 14.97	0.98	131.50 ± 14.37	130.38 ± 16.61	0.49	131.24 ± 14.52	132.13 ± 15.74	0.54
DBP (mmHg)	77.53 ± 9.21	77.67 ± 9.06	77.18 ± 9.59	0.56	77.43 ± 9.02	78.24 ± 10.43	0.47	77.62 ± 9.11	76.90 ± 9.87	0.55

Data were presented as mean ± SD; *P < 0.05, **P < 0.01.

BMI: body mass index; GPT: glutamic pyruvic transaminase; GFR: glomerular filtration rate; HbA1c: glycosylated hemoglobin; HDL: high density lipoprotein; LDL: low density lipoprotein; SBP: systolic blood pressure; DBP: diastolic blood pressure.

**Table 2:** Comparisons of domains of SF-36 and DIMS between constitutions of Yin-Xu, Yang-Xu, and Stasis^#^.

Variable	Yin-Xu		*F* value	Yang-Xu		*F* value	Stasis		*F* value
	No (*n* = 509)	Yes (*n* = 196)		No (*n* = 614)	Yes (*n* = 91)		No (*n* = 615)	Yes (*n* = 90)	
SF36									
PF	82.25 ± 18.07	69.44 ± 23.00	49.14∗∗∗	80.89 ± 18.83	63.85 ± 24.01	41.99∗∗∗	80.90 ± 18.92	63.61 ± 23.45	44.62∗∗∗
RP	74.51 ± 34.61	52.55 ± 42.06	42.38∗∗∗	71.90 ± 36.05	44.78 ± 43.06	32.72∗∗∗	72.72 ± 35.77	38.89 ± 40.56	67.73∗∗∗
BP	83.46 ± 19.28	68.71 ± 22.92	63.84∗∗∗	81.68 ± 20.09	63.71 ± 23.41	48.30∗∗∗	81.84 ± 20.38	62.39 ± 20.54	71.40∗∗∗
GH	58.69 ± 20.75	43.18 ± 23.41	66.10∗∗∗	56.74 ± 21.73	38.42 ± 21.99	56.10∗∗∗	57.19 ± 21.39	35.12 ± 21.29	83.72∗∗∗
VT	59.56 ± 11.44	52.04 ± 12.33	58.37∗∗∗	58.77 ± 11.66	48.68 ± 11.88	58.98∗∗∗	59.10 ± 11.42	46.28 ± 11.18	99.60∗∗∗
SF	82.63 ± 18.85	72.13 ± 23.46	31.47∗∗∗	81.50 ± 19.81	67.58 ± 22.97	30.14∗∗∗	82.04 ± 19.06	63.75 ± 24.65	45.56∗∗∗
RE	94.42 ± 17.69	81.29 ± 32.05	29.48∗∗∗	92.66 ± 20.84	78.02 ± 33.41	16.48∗∗	93.11 ± 20.00	74.81 ± 35.44	22.94∗∗∗
MH	74.47 ± 10.77	68.59 ± 15.25	24.40∗∗∗	73.97 ± 11.83	65.19 ± 13.86	32.95∗∗∗	74.20 ± 11.51	63.56 ± 14.61	43.82∗∗∗
PCS	47.55 ± 9.21	40.40 ± 11.10	64.32∗∗∗	46.70 ± 9.58	37.87 ± 11.46	48.86∗∗∗	46.82 ± 9.52	37.00 ± 11.14	63.20∗∗∗
MCS	51.59 ± 6.28	48.97 ± 8.69	14.59∗∗	51.32 ± 6.84	47.80 ± 8.23	15.05∗∗	51.50 ± 6.56	46.53 ± 9.13	24.80∗∗∗
DIMS									
Scale 1	49.22 ± 4.84	43.06 ± 4.94	226.80∗∗∗	48.34 ± 5.16	41.90 ± 5.21	122.99∗∗∗	48.32 ± 5.23	41.97 ± 4.83	117.94∗∗∗
Scale 2	25.78 ± 4.03	22.71 ± 4.75	63.84∗∗∗	25.36 ± 4.23	21.97 ± 4.78	49.28∗∗∗	25.47 ± 4.14	21.18 ± 4.72	81.36∗∗∗
Scale 3	33.70 ± 4.21	31.99 ± 4.58	22.18∗∗∗	33.44 ± 4.32	31.78 ± 4.54	11.49∗∗	33.61 ± 4.24	30.61 ± 4.47	38.69∗∗∗
Scale 4	13.59 ± 3.42	12.02 ± 3.89	24.70∗∗∗	13.31 ± 3.58	12.07 ± 3.78	9.49∗	13.45 ± 3.50	11.12 ± 3.82	33.87∗∗∗

^#^Data were presented as mean ± SD; *P < 0.05; **P < 0.01; ***P < 0.001.

PF: physical function, RP: role physical, BP: bodily pain, GH: general health, VT: vitality, SF: social functioning, RE: role emotional, MH: mental health, PCS: physical component summary, and MCS: mental component summary.

DIMS: diabetes impact measurement scale, scale 1: symptoms, scale 2: diabetes-related morale, scale 3: social role fulfillment, and scale 4: well-being.

**Table 3:** The adjusted regression coefficients of Yin-Xu, Yang-Xu, and Stasis on health-related quality of life measured by SF-36^#^.

Variable	PF		RP		BP		GH		VT	
	*β* (SE)	Standardized *β*	*β* (SE)	Standardized *β*	*β* (SE)	Standardized *β*	*β* (SE)	Standardized *β*	*β* (SE)	Standardized *β*
Yin-Xu (no as reference group)										
Yes	−4.86 (1.58)∗∗	−0.11	−9.97 (3.38)∗∗	−0.12	−7.47 (1.90)∗∗∗	−0.16	−8.37 (2.02)∗∗∗	−0.17	−3.32 (1.09)∗∗	−0.12
Yang-Xu (no as reference group)										
Yes	−8.38 (2.19)∗∗∗	−0.14	−9.57 (4.69)∗	−0.09	−6.30 (2.64)∗	−0.10	−5.70 (2.80)∗	−0.08	−3.58 (1.51)∗	−0.10
Stasis (no as reference group)										
Yes	−5.69 (2.23)∗	−0.09	−15.26 (4.76)∗∗	−0.13	−8.00 (2.68)∗∗	−0.12	−11.32 (2.84)∗∗∗	−0.16	−7.94 (1.54)∗∗∗	−0.21
*R* ^²^	41%		23%		25%		24%		24%	

	SF		RE		MH		PCS		MCS	
	*β* (SE)	Standardized *β*	*β* (SE)	Standardized *β*	*β* (SE)	Standardized *β*	*β* (SE)	Standardized *β*	*β* (SE)	Standardized *β*

Yin-Xu (no as reference group)										
Yes	−3.82 (1.91)∗	−0.08	−8.45 (2.15)∗∗∗	−0.17	−2.49 (1.12)∗	−0.09	−3.22 (0.83)∗∗∗	−0.14	−1.39 (0.64)∗	−0.09
Yang-Xu (no as reference group)										
Yes	−4.79 (2.65)	−0.08	−2.15 (2.97)	−0.03	−2.04 (1.55)	−0.06	−3.76 (1.15)∗	−0.12	−0.32 (0.89)	−0.02
Stasis (no as reference group)										
Yes	−11.23 (2.69)∗∗∗	−0.18	−11.16 (3.02)∗∗	−0.16	−8.24 (1.57)∗∗∗	−0.22	−3.53 (1.17)∗	−0.11	−4.39 (0.90)∗∗∗	−0.20
*R* ^²^	20%		16%		23%		35%		22%	

(1) ^#^Parameter estimates (SE), *P < 0.05;**P < 0.01; ***P < 0.001.

(2) Parameter estimates were from multiple linear regression adjusting for age, height, weight, smoking, regular exercise, fasting glucose, duration of diabetes, glycosylated hemoglobin, triglycerides, GPT, LDL-C, creatinine, GFR, diabetic neuropathy, diabetic retinopathy, metabolic syndrome, and comorbidity.

(3) PF: physical function, RP: role physical, BP: bodily pain, GH: general health, VT: vitality, SF: social functioning, RE: role emotional, MH: mental health, PCS: physical component summary, and MCS: mental component summary.

**Table 4:** The adjusted regression coefficients of Yin-Xu, Yang-Xu, and Stasis on diabetics with the quality of life measured by diabetes impact measurement scales^#^.

Variable	Symptoms		Related morale		Social role		Well-being	
	*β* (SE)	Standardized *β*	*β* (SE)	Standardized *β*	*β* (SE)	Standardized *β*	*β* (SE)	Standardized *β*
Yin-Xu (no as reference group)								
Yes	−4.00 (0.42)∗∗∗	−0.32	−1.63 (0.40)∗∗∗	−0.16	−0.88 (0.37)∗	−0.09	−0.85 (0.33)∗∗	−0.11
Yang-Xu (no as reference group)								
Yes	−2.58 (0.58)∗∗∗	−0.15	−0.76 (0.56)	−0.06	−0.08 (0.52)	−0.01	−0.35 (0.46)	0.03
Stasis (no as reference group)								
Yes	−2.34 (0.59)∗∗∗	−0.14	−2.97 (0.57)∗∗∗	−0.22	−1.65 (0.53)∗∗	−0.12	−1.71 (0.47)	−0.15
*R* ^²^	45%		23%		30%		20%	

(1) ^#^Parameter estimates (SE).

(2) *P < 0.05; **P < 0.01; ***P < 0.001.

(3) Parameter estimates were from multiple linear regression adjusting for age, height, weight, smoking, physical activity, fasting glucose, duration of diabetes, glycosylated hemoglobin, triglycerides, GPT, LDL-C, creatinine, GFR, diabetic neuropathy, diabetic retinopathy, metabolic syndrome, and combined morbidity.
